# AT THE INTERSECTION OF CULTURE AND MENTAL HEALTH

**DOI:** 10.1093/geroni/igac059.1892

**Published:** 2022-12-20

**Authors:** Daniel Jimenez

**Affiliations:** University of Miami Miller School of Medicine, Miami, Florida, United States

## Abstract

Although mental health disorders affect people across the lifespan, older adults face unique issues associated with accessing mental health treatment. These structural and psychosocial challenges are further exacerbated among older racial and ethnic minorities. Often compared to their white counterparts, racial and ethnic minority older adults face specific cultural factors and other systemic barriers that create stark disparities in diagnosis, treatment, and access to care. Increasing research on identifying barriers to treatment for older racial and ethnic minority adults has been recognized as an integral component in enhancing treatment access to improve behavioral health outcomes among these marginalized groups. Therefore, this review article aims to investigate the intersection of mental health and culture through the lens of three racial and ethnic minority groups in the United States – Blacks/African-Americans, Asian Americans, and Latinos. Authors provide unique insights on the differing needs of these under-researched communities by exploring psychiatric comorbidity, experiences with seeking, accessing, and engaging in treatment, and the unique cultural and psychosocial factors that affect treatment outcomes for these diverse groups. Future directions and recommendations to provide appropriate mental healthcare to Black/African-American, Asian American, and Latino communities are discussed with special attention placed on cultural adaptations, models of care, prevention, and practical strategies that can be implemented to reduce disparities and increase health equity.

